# Comparison of Hadlock and INTERGROWTH-21st Growth Charts for Estimating Fetal Weight in the Third Trimester via Ultrasound

**DOI:** 10.7759/cureus.81333

**Published:** 2025-03-28

**Authors:** Rashi Saini, Sumitra Bachani, Jyotsna Suri, Monika Gupta, Aprajita Gupta, Poornima Sharma, Pradeep Debata

**Affiliations:** 1 Department of Obstetrics and Gynecology, Vardhman Mahavir Medical College and Safdarjung Hospital, New Delhi, IND; 2 Department of Pediatrics, Vardhman Mahavir Medical College and Safdarjung Hospital, New Delhi, IND

**Keywords:** estimated fetal weight, fetal biometry, fetal growth charts, fetal growth restriction, fetal growth velocity, hadlock's formula, intergrowth chart

## Abstract

Introduction

Early identification of growth abnormalities is crucial in preventing fetal death and ensuring optimal care for fetuses requiring close monitoring and timely delivery. Sonographic estimation of fetal weight must be quantified in centiles according to gestational age reference charts to assess fetal growth velocity and size. These measurements can categorize the fetus as small, appropriate, or large for gestational age. Recent discussions have highlighted the increasing need for evidence-based guidelines for selecting the most suitable growth charts.

Objectives

The objective of this study is to compare the diagnostic accuracy of Hadlock versus INTERGROWTH 21st (IG 21) growth charts for estimating fetal weight.

Methods

This is a prospective cohort study conducted on 277 pregnant women with singleton fetuses at 32-34 weeks of gestation in the Department of Obstetrics and Gynecology at a tertiary care referral center in North India. The participants underwent two ultrasound scans, first at the 32- to 34-week period of gestation (POG) and then a second ultrasound scan at the 38- to 40-week POG or one week before delivery. Labor and delivery outcomes were noted, and neonatal weight was recorded. The mother and neonate were observed for any perinatal complications till discharge from the hospital.

Results

The mean POG at the first scan was 33.00 weeks. At the first scan, the mean of estimated fetal weight (EFW) by Hadlock was 1966.96 g. The mean of EFW by IG 21 was 1861.50 g. There was a strong positive correlation between EFW by IG 21 and EFW by Hadlock in the first scan, and this correlation was statistically significant (r = 0.78, p ≤ 0.001). The mean POG at the second scan was 38.63 weeks. The mean of EFW in the second scan by Hadlock was 2905.24 g. The mean of EFW in the second scan by IG 21 was 2829.32 g. There was a strong positive correlation between EFW by IG 21 and EFW by Hadlock in the second scan, and it was statistically significant (r = 0.71, p ≤ 0.001). For every 1 g increment in EFW on the second scan by Hadlock, the birth weight (g) increases by 0.86 g. For every 1 g increment in EFW on the second scan by IG 21, the birth weight increases by 0.82 g.

Conclusion

Both Hadlock and IG 21 charts have a fair agreement with EFW in early and late trimester scans. Hence, either one of the two can be used for fetal biometry.

## Introduction

The prenatal diagnosis of abnormal fetal growth patterns, such as growth retardation and growth acceleration, is vital since, in utero, recognition of these patterns can reduce the perinatal morbidity and mortality associated with their occurrence. The World Health Organization (WHO) defines the perinatal period as between 22 completed weeks of pregnancy and seven days after birth. India's current perinatal mortality rate is 26 (2013) [1], and the neonatal mortality rate (NMR-2019) is 21.66 per 1000 [2]. Early detection of growth abnormalities can prevent fetal demise and identify fetuses requiring intensive monitoring and timed delivery, thus improving antenatal care [3].

Ultrasound technology has revolutionized medical diagnostics, emerging as a pivotal tool across various fields. In gynecology, it provides critical insights into reproductive health, aiding in assessing ovarian and uterine conditions. Later, ultrasound became indispensable in obstetrics, offering a window into prenatal development and ensuring the well-being of both mother and fetus. This non-invasive, real-time imaging technique continues to bridge the gap between patient care and advanced medical precision.

Ultrasound biometry is an essential clinical tool for identifying, monitoring, and managing fetal growth restriction [3]. The commonly measured fetal biometric parameters are biparietal diameter (BPD), head circumference (HC), abdominal circumference (AC), and femur diaphysis length (FL). These biometric measurements can be used to calculate the estimated fetal weight (EFW) using different formulae [4-6].

The EFW calculated on sonography has to be quantified in centiles as per reference charts related to the period of gestation (POG) to determine the growth velocity and size of the fetus. This can be classified as small, appropriate, and large for a gestational fetus. Various growth charts, such as the WHO, INTERGROWTH 21st (IG 21), Hadlock, and customized growth charts, are available for reference. The IG 21 and the WHO employed a similar method [7,8]. IG 21 was a multicenter, multiethnic, population-based project conducted between 2009 and 2014 in eight countries. The project's main goal was to examine growth, health, nutrition, and neurodevelopment from less than 14 weeks of gestation up to two years of age [9]. It followed the same conceptual framework as the WHO Multicenter Growth Reference Study, aiming to create global standards for pregnancy dating, maternal weight gain, fetal growth, newborn size, the postnatal development of preterm infants, and cognitive development by two years of age.

There is an increasing need for more evidence to guide recommendations on which growth charts to use. Ideally, we should compare the diagnostic accuracy and misclassification rates of small for gestational age and large for gestational age fetuses regarding their morbidity and mortality using different criteria. This comparison is necessary to make informed recommendations and address a significant data gap [10].

Previous studies have linked EFW centiles from these charts with adverse outcomes but only evaluated the chart’s predictive ability at the 10th or 5th centiles. This is an important limitation because the new charts may need more extreme cut-off points since they reflect growth patterns under optimal conditions [10]. Currently, for the Indian population, there is no established single reference chart that can be used for estimating fetal weight and growth velocity and predicting associated perinatal outcomes [4]. The current study aims to compare the diagnostic accuracy of Hadlock versus IG 21st growth charts for estimating fetal weight.

## Materials and methods

**Figure 1:**
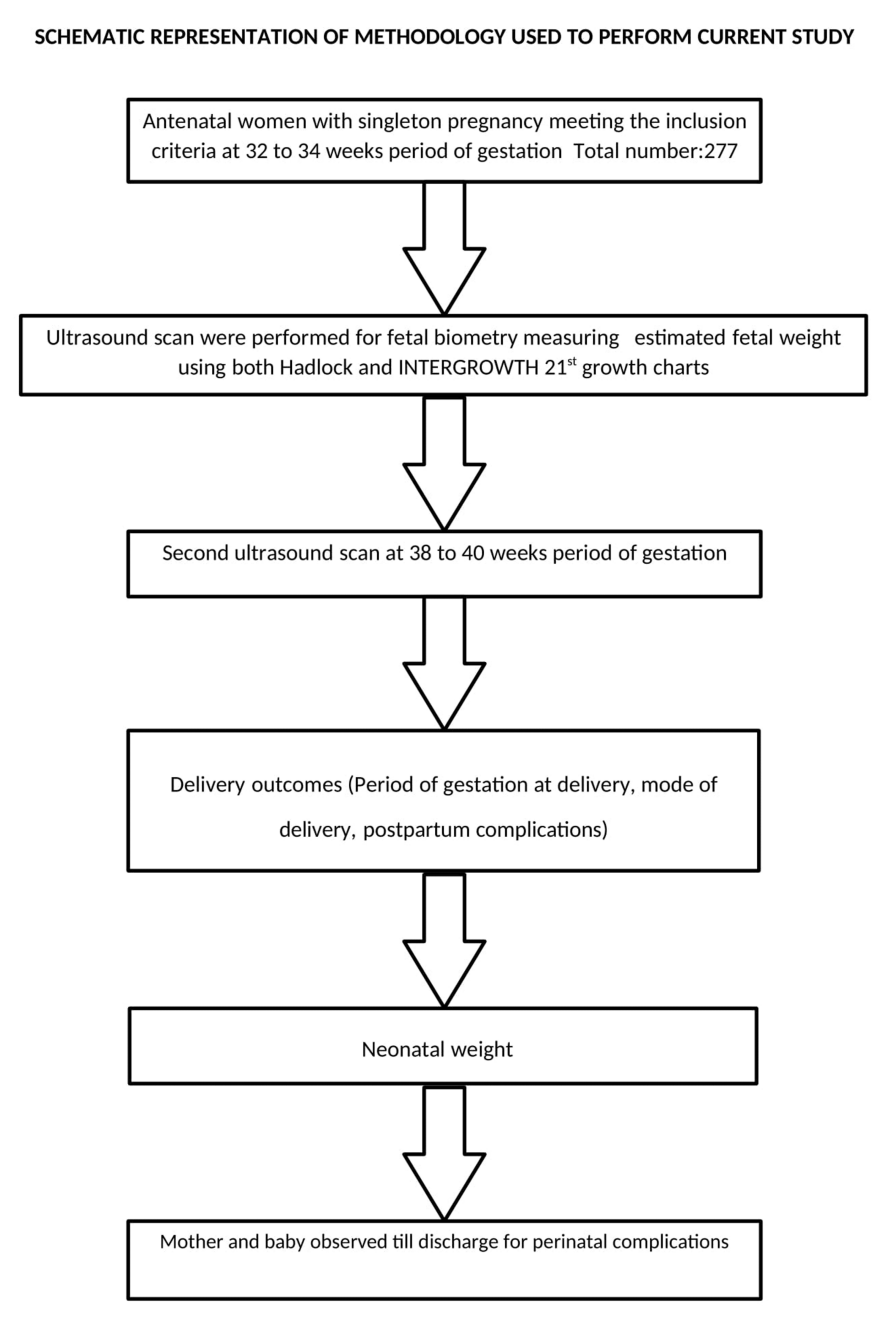
Study flow

This prospective cohort study was conducted on 277 pregnant North Indian women with singleton fetuses at 32-34 weeks of gestation in the Department of Obstetrics and Gynecology at Vardhman Mahavir Medical College and Safdarjung Hospital, New Delhi, India. The inclusion criteria included women with live singleton pregnancies at 32-34 weeks with no high-risk factors such as gestational hypertension, preeclampsia, diabetes mellitus, or any chronic medical illness, and without any gross congenital anomalies in the fetus. The gestational age was calculated as per the American College of Obstetricians and Gynecologists (ACOG) Committee Opinion No. 700 [11]. All women with singleton pregnancies at 32-34 weeks of gestation who met the inclusion criteria were included in the study after informed consent. All pregnant women who delivered before performing the second scan, intrauterine fetal death, fetuses with gross congenital anomalies, and women with pre-eclampsia or eclampsia, gestational diabetes mellitus, and intrahepatic cholestasis of pregnancy (IHCP) were excluded from the study. The study took approval from the Institutional Ethics Committee, Vardhman Mahavir Medical College and Safdarjung Hospital, New Delhi (06/2022/CC-163). The participants underwent two ultrasound scans, first at 32 to 34 weeks of gestation and then a second ultrasound scan at 38 to 40 weeks of gestation or one week before delivery. The ultrasound was performed as per the International Society of Ultrasound in Obstetrics and Gynecology (ISUOG) practice guidelines [4]. The ultrasounds were performed by a single sonographer with 30 years of experience with the Philips Affiniti 50 Ultrasound system (Philips, Netherlands). Women were treated according to institutional obstetric protocols. Labor and delivery outcomes were noted, and neonatal weight was recorded. The mother and neonate were observed for any perinatal complications till discharge from the hospital (Figure 1).

Taking the results from the study by Cheng et al. [12] as a reference, the minimum required sample size with a 3% margin of error and a 5% level of significance is 249 patients. To reduce the margin of error, the total sample size taken is 280. A p-value of less than 0.05 was considered statistically significant. Taking lost to follow up as 10%, n ≥ 249/.9 ≥ 276.67 = 277 (approximately).

Categorical variables were reported as numbers and percentages (%), while continuous variables were expressed as mean ± SD and median. The normality of the data was assessed using the Kolmogorov-Smirnov test. If the normality is rejected, then nonparametric tests are used. Statistical tests were applied. Quantitative variables were associated with the outcome using an independent t-test/Mann-Whitney test (when the data sets were not normally distributed). A Bland-Altman plot was used to compare EFW and birth weight. Analysis was done using IBM SPSS Statistics for Windows, Version 21 (Released 2012; IBM Corp., Armonk, New York, United States).

## Results

The mean age of the study population was 25.49 ± 3.51 years. The maximum number of participants (154, 55.6%) were between the age group of 18-25 years. Amongst 277 women, 127 (45.8%) were primigravida and 150 (54.2%) were multigravida (Table 1).

**Table 1:** Demographic characteristics of study population POG: period of gestation; SD: standard deviation

Clinical details	Mean ± SD	
Age (years)	25.49 ± 3.51	
Age group	Number of women	Percentage (%)
18-25 years	154	55.6
26-30 years	94	33.9
>30 years	29	10.5
Gravidity		
Primigravida	127	45.8
Multigravida	150	54.2
Mean POG at first scan	33.00 weeks	
Mean POG at second scan	38.63 weeks	

The first scan was done at 32 to 34 weeks POG. The mean POG at the first scan was 33.00 weeks. At the first scan, the mean of EFW by Hadlock was 1966.96 g. The mean of EFW by IG 21 was 1861.50 g. There was a strong positive correlation between EFW by IG 21 and EFW by Hadlock in the first scan, which was statistically significant (r = 0.78, p ≤ 0.001).

The mean POG at the second scan was 38.63 weeks. The mean of EFW in the second scan by Hadlock was 2905.24 g. The mean of EFW in the second scan by IG 21 was 2829.32 g. There was a strong positive correlation between EFW by IG 21 and EFW by Hadlock in the second scan, which was statistically significant (r = 0.71, p ≤ 0.001) (Table 2).

**Table 2:** Association between EFW by IG 21 and EFW by Hadlock at first scan and second scan EFW: estimated fetal weight; IG 21: INTERGROWTH 21st A p-value less than 0.05 is considered significant; * indicates significant p-values

Correlation	Pearson's correlation coefficient (r)	p-value
EFW (first scan) (IG 21) vs. EFW (first scan) (Hadlock)	0.78	<0.001*
EFW (second scan) (IG 21) vs. EFW (second scan) (Hadlock)	0.71	<0.001*

**Figure 2:**
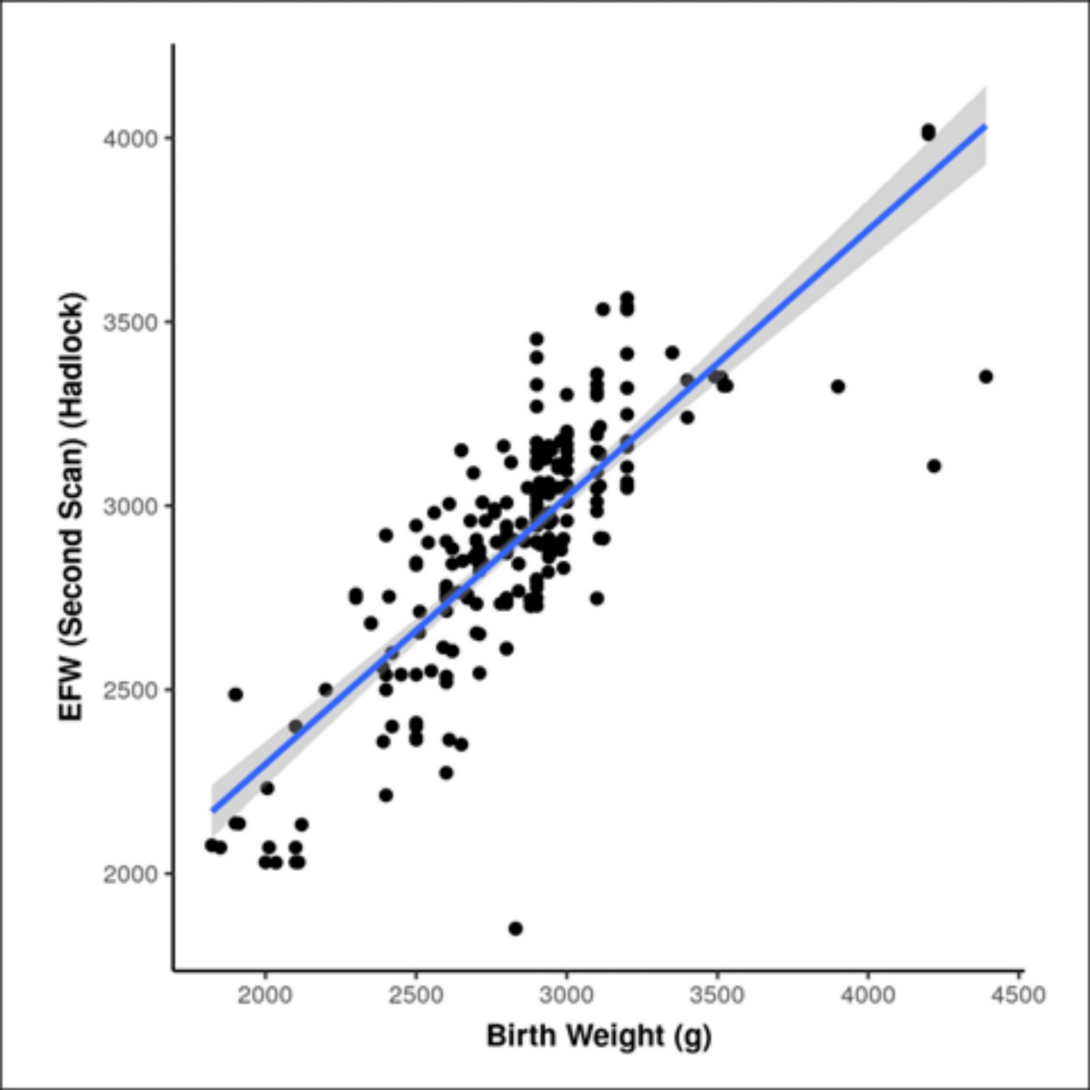
Correlation of EFW by Hadlock in second scan and birth weight EFW: estimated fetal weight

There was a strong positive correlation between birth weight and EFW by Hadlock in the second scan and this correlation was statistically significant (r = 0.8, p ≤ 0.001) (Figure 2). For every 1 g rise in EFW on the second scan by Hadlock, the birth weight (g) increases by 0.86 g.

**Figure 3:**
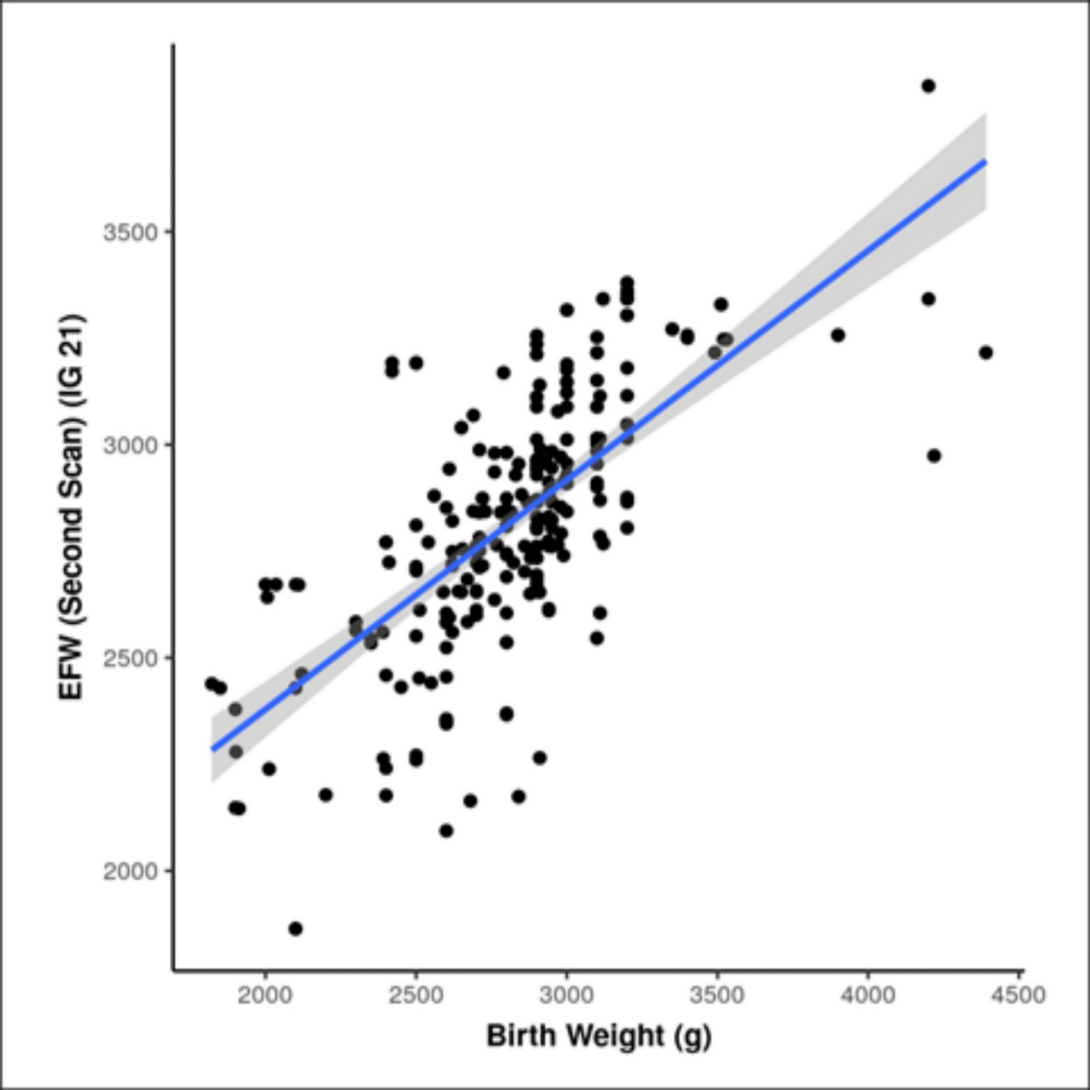
Correlation of EFW by IG 21 in second scan and birth weight EFW: estimated fetal weight; IG 21: INTERGROWTH 21st

There was a strong positive correlation between birth weight and EFW by IG 21 in the second scan, and this correlation was statistically significant (r = 0.67, p ≤ 0.001) (Figure 3). For every 1 g rise in EFW on the second scan by IG 21, the birth weight increases by 0.82 g (Table 3).

**Table 3:** Association between birth weight and EFW by Hadlock and IG 21 in second scan EFW: estimated fetal weight; IG 21: INTERGROWTH 21st The p-value less than 0.05 is considered significant; * indicates significant p-values

Correlation	Spearman correlation coefficient (r)	p-value
Birth weight (g) vs. EFW (second scan) (Hadlock)	0.8	<0.001*
Birth weight (g) vs. EFW (second scan) (IG 21)	0.67	<0.001*

The mean of POG at delivery was 39.2 weeks. All pregnant women delivered within two weeks of performing the second scan, and most of the women (228 women, 82.31%) delivered within a week of performing the second scan. Spontaneous onset of labor occurred in 67.7% of the participants, and 32.3% of the participants underwent induction. The most common indication for induction was decreased fetal movements. Amongst the study population, 228 (82.6%) of the population delivered vaginally, followed by emergency cesarean section in 27 (9.8%). The mean birth weight was 2836.48 g. Most (62.1%) of neonates had birth weights between 2.5 and 3 kg. Amongst the neonates, 15 of 277 (5.48%) babies needed admission to the neonatal intensive care unit (NICU). Low birth weight was the most common indication, comprising seven neonates for NICU admission.

## Discussion

Birthweight that reflects intrauterine growth is an essential determinant of perinatal morbidity and death [3,13]. Appropriate interventions in the high-risk group can reduce adverse perinatal outcomes [14]. Estimating fetal weight is pivotal in monitoring fetal growth and holds significance in forecasting neonatal outcomes [15-17].

Considerable research endeavors have been dedicated to identifying the most precise formula for estimating EFW. Within the clinical framework of the United Kingdom, the Royal College of Obstetricians and Gynaecologists (RCOG) advocates for the employment of the Hadlock A formula (Hadlock 1985 formula), a regression-based methodology integrating all three primary fetal measurements (AC, BPD, and FL). While the preponderance of evidence substantiates the superiority of formulas encompassing all three parameters, AC emerges as the most sensitive individual predictor of aberrant fetal growth [18]. The Hadlock regression model (Hadlock I) used in the present study utilizes four fetal measurements of HC, BPD, AC, and FL, which are considered reliable and widely used.

Grantz et al. conducted a comparison of the Eunice Kennedy Shriver National Institute of Child Health and Human Development Fetal Growth Studies, focusing on interracial differences in EFW. They evaluated the findings from IG 21 and the WHO Multicenter Growth Reference Study. The study concluded that the 50th percentile EFW for IG 21 was smaller than the 50th percentile EFW for the WHO. At 39 weeks of gestation, the EFW at the 50th percentile was as follows: 3,502 g for white infants, 3,330 g for Hispanic infants, 3,263 g for Asian infants, and 3,256 g for Black infants, according to the National Institute of Child Health and Human Development (NICHD) Study. In comparison, the EFW at the 50th percentile was 3,186 g for the IG 21 and 3,403 g for the WHO [7,19]. In the current study, the 50th percentile for EFW was 2912 g for Hadlock, and the 50th percentile for EFW was 2841 g for IG 21. Hence, assessing fetal growth using a single measurement continues to be the standard clinical practice, even though it is understood that this one-time measurement only indicates size. To estimate growth trajectory accurately, at least two measurements taken at different times are necessary. One of the key contributions of prospective studies in this area will be the ability to assess fetal growth velocity [7]. In the current study, all the participating 277 women with singleton pregnancies have undergone two ultrasound scans.

Monier et al. conducted a retrospective study in women who delivered between 22 and 34 weeks of gestation, including 578 women, with information available on ultrasound measurements and ultrasound to the delivery time of less than two days. However, in the current study, the ultrasound was done in the late third trimester. They reported that the mean (SD) percentage errors for Hadlock and IG 21 differed significantly. EFW with the Hadlock formula was close to the mean birthweight across the gestational age spectrum, whereas EFW with the INTERGROWTH formula was lower than both Hadlock and the mean birthweight from 28 to 34 weeks. Differences were particularly marked between 29 and 34 weeks when the INTERGROWTH formula significantly underestimated fetal weight [20]. In the current study, the mean of POG at the first scan was 33.00 weeks, which is the early third trimester. At the first scan, there was a strong positive correlation between EFW by IG 21 and EFW by Hadlock, and this correlation was statistically significant. However, Monier et al. conducted scans between 22 and 34 weeks of gestation, which is a broad time period of 12 weeks, and in the second trimester, many fetal growth changes can happen, with maximum fetal weight gain in the third trimester.

A prospective cross-sectional cohort study involving 970 women who were more than 34 weeks pregnant aimed to evaluate the suitability of the IG 21 and WHO EFW references. The results indicated that EFW by the Hadlock prediction model had the lowest mean and standard deviation of absolute error. Moreover, the variations between the two predicted weights were unlikely to have any clinical relevance in the Chinese population [12], which was similar to the current study in the Indian Asian population.

Cheng et al. conducted a prospective cross-sectional cohort study in China, where viable singleton pregnancies at 11-13 weeks were randomized to undergo a standardized fetal biometric scan after 20 weeks. Population-specific EFW gestation references were constructed using the Hadlock and IG 21 formulas. Differences between predicted EFW on the day of delivery and birth weight for fetuses with biometry scans ≥34 weeks were assessed. They found that neither of the international EFW references (IG 21 and WHO) accurately reflected the local population and would require customization. Additionally, the WHO 10th, 50th, and 90th centiles of the EFW reference were consistently greater than the corresponding centiles found in the local population, despite both using the same Hadlock model for predicting fetal weight, and there is a marked difference in the fetal weight of their Chinese population and the IG 21 and WHO international standard and reference [12]. There was no statistically significant difference between fetal weight predicted by Hadlock and IG 21, each, and when compared to birth weight.

Certain limitations should be acknowledged to enhance the robustness of future research. Firstly, the subjects are primarily individuals of North Indian ethnicity, all of whom presented to the tertiary care center. Thus, the lack of diversity prevents the generalizability of the findings across different ethnic groups. A large multi-center study would improve the statistical power and capture a broader spectrum of clinical presentations, as the current study was limited to low-risk women. The study's strength lies in its prospective nature, which provides a robust methodological framework. A single-center and single observer and operator study minimizes the bias; it's a direct comparison of two distinct charts in the same cohort.

## Conclusions

Both Hadlock and IG 21 charts agree fairly with EFW in early and late trimester scans. Hence, either one of the two can be used for fetal biometry.
